# ‘She didn't know what to do with me’: The experience of seeking community mental health support after spinal cord injury

**DOI:** 10.1080/10790268.2025.2479957

**Published:** 2025-03-31

**Authors:** Katherine A. Finlay, Phoebe Brook-Rowland, Margaret Tilley

**Affiliations:** 1 School of Psychology and Clinical Language Sciences, University of Reading, Reading, UK; 2 Faculty of Education and Sport, University of Bedfordshire, Luton, UK; 3 School of Psychology, University of Buckingham, Buckingham, UK

**Keywords:** Qualitative, Cognitive behavioral therapy, Treatment failure, Rehabilitation, Psychological wellbeing

## Abstract

**Context/Objectives:**

Adults with spinal cord injury in the UK do not currently have specialized access to SCI-informed community-based mental health support, despite their elevated risk of mental health decline. The lack of SCI-informed therapeutic support may increase the likelihood of mental health treatment failure. This study sought to qualitatively explore the experience of accessing, or attempting to access, generic (non-SCI-informed) mental health support when living with a spinal cord injury.

**Design:**

Qualitative, exploratory study using thematic analysis.

**Setting:**

Community-based sample in the UK.

**Participants:**

Twenty people with spinal cord injury (10 female, 10 male) were recruited from a UK-based, SCI-specific support charity.

**Interventions:**

Semi-structured interviews (mean length = 83 min, SD = 13.5 min).

**Outcome Measures:**

9-item semi-structured interview schedule, addressing mental health service use.

**Results:**

Three themes were identified: (1) Therapeutic timeliness; (2) A disconnect with standard services; and (3) Successful systems for support. The inpatient-to-outpatient transition represents a critical time window during which mental health is vulnerable to decline, requiring responsive access to mental health services throughout the lifespan. The lack of tailored, SCI-informed mental health services inhibits therapeutic engagement and limits perceived treatment outcomes.

**Conclusions:**

Without SCI-informed care, generic mental health service referrals risk early termination of support and treatment disengagement. Mental health treatment withdrawal is initiated by both patients and their allocated healthcare professionals. This study demonstrates an evident need to develop programs for people with SCI to train as (peer) mental health practitioners, and to develop SCI-specific training modules for mental health care practitioners.

## Introduction

Spinal cord injury (SCI) is an irrefutably life-altering, long-term condition associated with life-long physical (1,2) and mental health implications (3). Within the SCI population, it is estimated around 22% of people struggle with depression, 27% with anxiety and between 11% and 17% with post-traumatic stress disorder (PTSD) (4–7). Research has focused on the initial acceptance of SCI, noting suicidal ideation is highest in the first two years post-injury (8,9), but mental wellbeing commonly remains problematic beyond this time period (10). Though not all people with SCI experience episodes of mental health decline, rates are commonly higher than those seen in people without SCI (11). Certainly, the combined impact of physical health difficulties and adjusting to a lifechanging injury/illness means that some people living with SCI (PLwSCI) may require significant support to avoid deterioration in their mental health (8,12).

In the UK, National Health Service (NHS) specialist Spinal Cord Injury Centre (SCIC) rehabilitation settings provide inpatients with mental health support via an initial mental health assessment and the inclusion of a Clinical Psychologist as an integral part of the SCIC Multidisciplinary Team (MDT) (13). Mental health is factored into inpatient rehabilitation goals and discharge planning (14). For outpatients, however, the psychological support profile is very different; unlike the regular MDT support received by SCIC inpatients, PLwSCI in the community receive much lower levels of interaction with psychological services (15): formal mental health assessments are not required and individuals with mental health concerns are most likely to be signposted to community services (16), despite clinical practice guidelines for Healthcare Professionals highlighting the importance of post-SCI mental healthcare (17). Currently, in the UK, people with mental health difficulties with or without SCI, are referred to Improving Access to Psychological Therapies (IAPT; NICE CG91). Yet, service evaluations increasingly have noted that community mental health services may be inadequate, showing waning efficacy for people with long-term conditions (18–20). The standardized mental health care pathways used for people in the general population may not be appropriate for PLwSCI. There may need to be service adaptation and healthcare professional training specific to SCI, if sustained mental health improvements are to be achieved.

### Aim

This study aimed to explore, in depth, the qualitative experiences of PLwSCI receiving standardized (condition non-specific) mental health care, delivered through community mental health care pathways.

## Materials and methods

### Design

Semi-structured interviews were analyzed via thematic analysis (TA) in combination with the creation of a ‘living codebook’ (21,22). TA is favored for applied health research as it is analytically robust and allows for data to be presented in an easily accessible format (23). Supplementing TA with a living codebook facilitates transparency and replicability in qualitative research (following 21,22). The living codebook enhances rigor in qualitative studies, allowing for a processual database to be maintained that accounts for codes, key terms and memos and documentation of the rationale behind unmatched codes (22,24). Creating a codebook during thematic analysis is increasingly used in medical qualitative research to facilitate investigations into programs or services (25,26) This study is reported using the 32-item COREQ-32 consolidated criteria for reporting qualitative research (27).

### Participants

Purposive sampling was used to recruit 20 participants (10 females, 10 males) from the Spinal Injuries Association, a charitable organization for people living with SCI in the United Kingdom. Inclusion criteria were: PLwSCI in the outpatient community who had accessed (or attempted to access) generic mental health services in the past 12 months. Purposive sampling was employed to ensure all levels of injury (cervical, thoracic, lumbar and sacral) were represented. Exclusion criteria were: participants reporting back/chronic pain but without formally diagnosed SCI; or those who had not used or attempted to use community mental health services (Table 1).

**Table 1:** Demographic details of participants with spinal cord injury.

Pseudonym	Age	Sex	Marital Status	Ethnicity	Employment Status	Years Since Injury	Injury Type	Level of Injury	Cause of Injury	Prior Mental Health Diagnosis	Currently Receiving Treatment
Scully	47	Male	Single	Mixed/Multiple ethnic groups	Long-term sick or disabled	26	Complete tetraplegia	Cervical	Sport/recreational activity	Depression Anxiety	Online mental health practitioner and anti-depressants
Florence	48	Female	Married	White Other	Volunteering	2	Complete paraplegia	Thoracic	Sport/recreational activity	No	No
John	47	Male	Cohabiting	White British	Volunteering	21	Complete tetraplegia	Cervical	Sport/recreational activity	No	Counseling
David	52	Male	Divorced/Separated	White British	Working as an employee full time	30	Complete paraplegia	Thoracic	Road traffic accident	No	No
Sarah	51	Female	Married	White British	Volunteering	5	Incomplete tetraplegia	Cervical	Illness	No	No
Malcolm	85	Male	Married	White British	Retired	18	Incomplete tetraplegia	Cervical	Sport/recreational activity	No	No
Louise	60	Female	Divorced/Separated	White British	Working as an employee full time	<1	Incomplete paraplegia	Lumbar	Other	No	No
Janet	61	Female	Single	White British	Retired	60	Incomplete paraplegia	Thoracic	Illness	No	No
Simon	40	Male	Divorced/Separated	White British	Long-term sick or disabled	17	Incomplete paraplegia	Sacral	Unknown	Depression	Cognitive behavioral therapy for pain and anti-depressants
Thomas	51	Male	Single	White British	Self-employed or freelance part-time	11	Incomplete paraplegia	Lumbar	Sport/recreational activity	No	No
Michael	48	Male	Single	White British	Self-employed or freelance part-time	26	Complete paraplegia	Thoracic	Falls	No	No
Jessica	63	Female	Married	White British	Furloughed	3	Complete paraplegia	Thoracic	Spinal Cord Stroke	No	No
Toby	37	Male	Married	White British	Working as an employee part-time	6	Complete paraplegia	Thoracic	Sport/recreational activity	Eating Disorder	No
Tim	56	Male	Married	White British	Long-term sick or disabled	9	Incomplete tetraplegia	Cervical	Falls	Depression	Adult mental health support (very occasional telephone conversations with Psychiatrist)
Beth	57	Female	Married	White British	Working as an employee full time	23	Incomplete paraplegia	Lumbar	Road traffic accident	No	No
Rachel	68	Female	Married	White British	Retired	8	Complete tetraplegia	Cervical	Falls	No	No
Sandra	56	Female	Married	White British	Furloughed	33	Incomplete paraplegia	Thoracic	Cavernoma bleed and removal.	No	No
Zoe	51	Female	Single	White British	Long-term sick or disabled	10	Incomplete tetraplegia	Cervical	Illness	No	Telephone counseling
Audre	52	Female	Single	White British	Long-term sick or disabled	8	Incomplete tetraplegia	Cervical	Suicide attempt	Paranoid Schizophrenia	No
Ian	64	Male	Married	White British	Long-term sick or disabled	1	Incomplete tetraplegia	Cervical	Illness	No	No

### Measures

A nine-item interview schedule was used (Table 2). Questions reviewed barriers and facilitators to improving mental wellbeing, motivations to seek community mental health support, and experiences of accessing/engaging with generic mental health services.

**Table 2:** Interview schedule.

*1.*	Can you tell me about what has happened to your mental health since you had your Spinal Cord Injury?
*2.*	What do you think are the core priorities for improving mental health for people with SCI as a whole?
*3.*	What do you think are the major barriers to improving mental health in people living with SCI?
*4.*	What do you think helps improve mental health for people living with SCI?
*5.*	Can you tell me about some of the mental health service provisions you know about?
*6.*	Can you tell me about your experience of deciding to seek help (or not to seek help) for your mental health?
*7.*	Can you tell me about the mental health services that you have used, if any, since your SCI?
*8.*	Can you tell me about what you think might have stopped you from using mental health services?
*9.*	Is there anything about SCI mental health and mental health services that we’ve not discussed that you would like to tell me more about?

### Procedure

Interviews were conducted via zoom (*n* = 17) or telephone call (*n* = 3). Prior to interviews, participants gave e-consent via the survey tool REDCap. Interviews lasted between 62 and 124 min (*M* = 83.2, SD = 13.45) and were audio-recorded and transcribed via otter.ai before transcripts were reviewed and quality audited. The semi-structured nature of the interview schedule allowed researchers to respond to areas of interest or concern which participants raised and probe responses (28). All interviews were conducted by the second author [PB-R], who holds advanced qualitative research methods training and postgraduate qualifications in Health Psychology. A rigorous, multi-stage analytic process was followed, working with a living codebook (following 21; see Supplementary Material 1 for analytic steps). All transcripts were double coded.

### Ethical consideration

The study gained ethical approval from the University of Reading Research Ethics Committee. Participants gave informed consent for interview recording and reproduction of anonymized quotes. Participants had the right to withdraw at any time and could elect to stop or pause the interview at any point. All personally identifiable data was removed at transcription and participant transcripts were pseudonymized to maintain confidentiality and anonymity.

## Results

**Figure 1:**
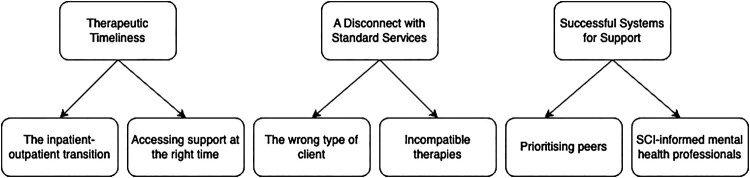
Thematic map of experiences accessing community mental health services after SCI.

Three main themes were evident (see Fig. 1): (1) *Therapeutic timeliness;* (2) *A disconnect with standard services*; and (3) *Successful systems for support*.

### Main theme 1: therapeutic timeliness

Participants highlighted that comprehending a life-altering injury is an *ongoing process* that needs long-term mental health support over time rather than short-term intervention. The transition to living at home was a focal point, however open-ended, long-term mental health changes suggested that readiness for treatment did not solely occur during the early, acute phase.

#### The inpatient-outpatient transition

Participants highlighted the enormity of the challenge of adapting to life post-discharge and the disparity in psychological support receipt when moving between inpatient and outpatient services: ‘When you get home, we had nothing. It all stops. You're supposed to just get on with life really. And I was shocked, to be honest’ (Rachel, 68, Complete Tetraplegia). The loss of connection with inpatient therapeutic support services was highly salient: ‘You come home and you're just isolated and that's when you really need the help. I have had absolutely no mental health help since I left’ (Jessica, 63, Complete Paraplegia). Moving from an SCI-informed environment, back into generic community services, exacerbated the mental health challenges experienced by PLwSCI: ‘I feel like … if you are going to be released from a spinal unit, that you should have something in the community that's already set up to kind of help you make that transition from a mental health standpoint’ (Zoe, 51, Incomplete Tetraplegia). It was felt that services did not support this transition:

It feels that there is no umbrella approach, there is no connected approach for mental health. And that's fine for short-term patients, but a spinal patient is seeing you for life. (Tim, 56, Incomplete Tetraplegia)

#### Accessing support at the right time

Community mental health support was difficult to access: ‘What has been so difficult is you have to go and ask for everything. You know, it's all self-referral now for mental health support. So, you can only ask for something … Throughout the time, I've struggled to find regular mental health support’ (Tim, 56, Incomplete Tetraplegia). Participants wished mental health support was proactive and explicitly and openly offered at regular intervals as part of ongoing post-discharge care for PLwSCI:

I was told it [the waiting list] would be six months or more, when I put my name down. The problem is, you don't want it tomorrow, next week, or a month away. I know it's not possible, but you need it now at that point, where you can't get out of that dip. You're constantly crying, and you try and try again. And then, you just go deeper in. And literally, there's no way out. (Rachel, 68, Complete Tetraplegia)

Readiness to talk about mental health does not come immediately after being injured, requiring SCI-informed mental health support outside of the inpatient setting:

So, for 10 years, I didn't [access mental health services]. It was quite a thing to reach out. And I probably could have reached out plenty of times before I got to the position that I did. And then it was so good when I did reach out. (John, 47, Complete Tetraplegia)

Participants expressed a two-directional ‘readiness’ for treatment: services need to be ‘ready’ to provide therapeutic support at the point that people are ‘ready’ for treatment:

Have the counsellors ready for when you’re ready, ‘cause you have to be ready yourself to see a counsellor, I think. (Sarah, 51, Incomplete Tetraplegia)

### Main theme 2: a disconnect with standard services

All participants were frustrated that accessing mental health support is not straightforward. Many PLwSCI who had successfully accessed standard mental health services withdrew themselves early as a result of perceived failure to support SCI-related mental health difficulties, with some finding services worsened their issues.

#### The wrong type of client

Participants felt that standard services were not equipped to deal with their issues: I've had people [Healthcare Professionals] talk to me about mental health, who have absolutely no idea what it's like to be left immobile, frightened to go out the house … for six years (Thomas, 51, Incomplete Paraplegia)*.* When SCI-specific knowledge was unavailable, in some cases the support was completely withdrawn: ‘They basically [Community mental health team] left it at “Oh, there's nothing we can do for you, we don’t know about spinal injuries, sorry, but there’s nothing we can do.” It’s not what you want to hear, when I needed help at that point’ (Scully, 47, Complete Tetraplegia). SCI-specific stressors were not understood by IAPT mental health practitioners and PLwSCI felt topics such as bladder and bowel management were taboo:

I think whatever NHS mental health service you'd go to talk to. They never cover or aren't willing to cover bowel and bladder. And that's one of the biggest issues that I think spinal patients suffer with mentally. It's the coming to terms with the bowel and bladder. (Sarah, 51, Incomplete Tetraplegia)

Due to therapists’ limited understanding of SCI, perceived stigma was exacerbated if the SCI was an incomplete injury:

I’m not disabled but I’m not able-bodied either, sort of floating in no man's land. It'd be nice if people just understood that, because people who are able-bodied think – well this is my opinion – people who are able-bodied see me as disabled and people who are disabled see me as able-bodied. So, I’m nothing. Sat in the middle on the fence. (Louise, 60, Incomplete Paraplegia)

Lack of knowledge of SCI on the part of the therapist, combined with feelings of ‘taboo subjects’ and stigma, meant that PLwSCI felt they were mismatched with community mental health services.

#### Incompatible therapies

Many felt that standard mental health services could only provide a ‘tickbox’ exercise due to the manualization of low- or moderate-intensity therapeutic approaches, lacking flexibility. When receiving manualized mental health support, participants felt unseen and unheard, often opting to terminate the support early.

They put me onto CBT [cognitive behavioural therapy], which I found completely useless. It just didn't help the situation I was in, or the problem I was going through. It just felt like it was a generalised way of sort of ticking boxes almost, that's what it felt like to me. (Toby, 37, Complete Paraplegia)

Consistently, Cognitive Behavioral Therapy (CBT), in particular, was noted as a therapeutic disconnect:

So, then they referred me for CBT. And I get there and all she would do was, you had to fill in whatever questionnaire about how you're coping, she would just look at that and spend 20 minutes looking at that. So, I gave up. I was getting absolutely nothing from it. (Florence, 48, Complete Paraplegia)

Standardized approaches were considered inappropriately for the magnitude of the challenges presented by living with SCI:

They did arrange someone to come and visit me and she was very efficient and sort of tried to look at the problems and find ‘appropriate’ solutions to the problems but it's not what you need at that time … they tried deep breathing with you and things like that [but] that's not going to enable you to go upstairs and read your child's story if you deep breathe is it, so I didn't find it very helpful at all. (Sarah, 51, Incomplete Tetraplegia)

Standardized approaches or manualization were therefore considered inappropriately tailored therapeutic approaches for mental wellbeing after SCI.

### Main theme 3: successful systems for support

PLwSCI reported the greatest mental health gains from speaking to someone who understands SCI. This took two forms: (i) speaking with peer mentors or friends with SCI, and (ii) speaking to SCI-informed practitioners or a counselor with SCI. This main theme highlighted that the individuals who are best able to support PLwSCI are those with prior knowledge of SCI, either experientially or through completing specialist training.

#### Prioritizing peers

The ideal scenario for community-based mental health support after SCI was to prioritize contact with peers with SCI for general support:

I had one really good [peer mentor]. She kind of mentored me … And she was the same as me, same level. And she had a tracheostomy and had to fight at times with the doctors and things. I kind of felt really relaxed with her. So, I could talk to her about things. (Rachel, 68, Complete Tetraplegia)

Such peer mentorship was also present informally through building new friendship networks: We [PLwSCI] support each other now through the good days and the bad days, so I think long term, you do need peer support (Sarah, 51, Incomplete Tetraplegia). Such peer support mentorship was consistently considered an invaluable mental health resources.

#### SCI-informed mental health professionals

Many felt that unless someone had experienced SCI or previously worked with SCI, they were unable to effectively use their therapeutic skillset or to provide realistic advice or guidance. In lieu, others sought support from private counselors who at least had prior experience working with chronic illnesses: I had to look around to see, number one, whether I could find a therapist that had dealt with people who are chronically ill. The only box that you can really tick for somebody like myself, I feel, is chronic illness (Zoe, 51, Incomplete Tetraplegia)

To understand the nature of SCI, knowledge of the lived experience of SCI was considered the minimum expectation when accepting a client with SCI:

I'm not saying they have to go and do a stint in the rehab centre or anything like that, but you know, a bit more research and background into it. And maybe get an idea by talking to actual patients who've lived through it or gone through it and can actually give a first-hand experience. (Toby, 37, Complete Paraplegia)

There was a strong desire to see more PLwSCI moving into mental health practitioner roles. Those who had been able to access a counselor who themselves had SCI spoke highly of the experience.

That was the first time I found someone that really understood what I was going through, and could talk to me about it, and ask me why I was doing certain things and really understand why I was doing those things and could empathise and talk me through things. It was the first time that I actually felt someone was ‘getting me’. (Sarah, 51, Incomplete Tetraplegia)

Adequate SCI-specific practitioner training was considered fundamental to develop a working therapeutic partnership.

## Discussion

The aim of this study was to explore the experience of accessing, or attempting to access, mental health services when living in the community with SCI. Thematic analysis identified three main themes: (1) *Therapeutic timeliness*; (2) *A disconnect with standard services*; and (3) *Successful systems for support*.

This study highlighted how the process of transitioning home after rehabilitation needs a more robust support system for protecting mental health in the immediacy. After discharge, PLwSCI report feeling that they are left on their own to cope and that there is an overriding need for more information on how to manage life post-discharge (29). Moving from an inpatient unit back into the community is accompanied by the negative mental health impact of losing the proximal peer support available in the ward environment, and being discharged from inpatient psychological care after transitioning out of hospital (30). This runs counter to the prevailing perception that discharge represents completion of the rehabilitation phase and returns a person with SCI (to some extent) to a more autonomous context. Provision of a coherent transition support intervention in the UK (31) may have the potential to mitigate the seismic impact of discharge on mental wellbeing.

This study further demonstrated that it is not only in the initial months after hospital discharge that PLwSCI need access to heightened and tailored mental health support. Comprehension of a life-altering injury continues long after transitioning home. Difficulties adjusting to life post-SCI have been well documented, often focusing on concepts such as acceptance (1,8). However, the current study indicates that for some, this process does not have a traditional endpoint. There is a need to step away from the dualistic assumption that individuals are either successful or unsuccessful in their adjustment after injury and instead there should be an increased focus on the fluctuating nature of mental health post-SCI (32). PLwSCI desire access to *open-ended mental health support*: having access to SCI-informed mental health support provisions immediately after injury is insufficient; care pathways need adaptation to enable PLwSCI to access SCI-informed support within the community at *any stage and any time.* These open-ended support needs may reflect the complex processes of prolonged adjustment, with PLwSCI oscillating between loss- and restoration-oriented grief in a regulatory process of active coping (33). In this way, desire to access mental health support at (multiple) timepoints other than solely in rehabilitation may be re-evaluated positively as evidence of ongoing and active adjustment.

The current study found that PLwSCI often struggle to access appropriate mental health support in the community and that standardized or manualized therapies are experienced as incompatible with SCI. Results from this study agreed with previous research that without a tailored approach, standard CBT administered to people with chronic health conditions leads to early termination (20,34), at times, worsens mental health outcomes (18), hardening PLwSCI to psychological interventions (35). This is contrary to other chronic conditions such as diabetes, COPD and CVD, where IAPT improved health outcomes (36), suggesting SCI warrants a more tailored approach. The mental health impact of changed bladder and bowel management can be significant, with perceptions of loss of control and independence (37). Mental health professionals who lack a comprehensive framework of understanding of the physical changes wrought by SCI further explains PLwSCI feel they are the ‘wrong type of client’. Equipping psychological practitioners with knowledge of common SCI stressors, experiences and presentations could allow them to better adapt CBT delivery and gain patient trust, reducing the likelihood of worsening mental health outcomes (34).

This study critically demonstrated the benefits of SCI-informed counseling and peer support structures. The benefits of peer support and peer mentors have been well documented (38), however it is of note that peer mentors are rarely trained in mental health and mental health improvements are therefore secondary outcomes in peer support interventions. Given the value that peer mentors already provide within SCI, further investigation is required as to whether providing peer mentors with mental health training would be beneficial, or rather looking to train PLwSCI solely in the provision of mental health support. This could widen the accessibility of mental health support for PLwSCI, thereby allowing individuals to access support at a time which is right for them.

### Limitations and future research

Though robust mitigation against bias was employed by sense checking with PLwSCI and the Spinal Injuries Association research board members, future studies could further embed PLwSCI in the research team. The results of this study are UK-specific (via IAPT) but may be extrapolated beyond this national context as they model wider research confirming widespread (international) dissatisfaction with generic mental health care (9). There is a need for a cross-national service evaluation to quantify the long-term therapeutic effects of standardized (non-SCI-specific) CBT for PLwSCI. Alternative therapeutic interventions such as SCI-informed Acceptance and Commitment Therapy should also be accessible for PLwSCI in community (39), providing an alternative to IAPT’s reliance on manualized CBT. Additionally, analysis of the confidence and capability of mental health practitioners should be assessed when working with PLwSCI, with the intention of creating additional SCI-informed training. The specific and deliberate inclusion of mental health assessments and the forging of closer links between SCICs and community psychiatric support services may be avenues for exploration.

## Conclusion

Proactively offering access to mental health support for PLwSCI when transitioning back into community care, represents a key mitigation against risk of psychological distress during this critical time window. For the subset of people with post-SCI mental health decline, mental health needs are heightened and ongoing for people living long-term with SCI and do not end with discharge to outpatient care. Combined with ageing and physical stressors such as bladder and bowel management, mental health needs typically increase. This study demonstrated the significant extent to which standardized mental health services are unable to provide adequate SCI-informed care, leading to early treatment withdrawal and worsening mental wellbeing. There is an urgent need to increase access, whether that be by training PLwSCI to become practitioners themselves, or by developing high-level Continuing Professional Development programs which would allow non-SCI practitioners to upskill their knowledge and clinical formulations, to dynamically encompass the implications of living SCI or chronic illness. Not everybody who incurs SCI will need mental health support but for those that do it is paramount they can access appropriate, responsive, inclusive, proactive and tailored mental health care.

## Supplementary Material

Supplementary Material 1.docx

## Data Availability

The data that support the findings of this study are available from the corresponding author upon reasonable request.
