# Supplementary material for: ‘She didn't know what to do with me’: The experience of seeking community mental health support after spinal cord injury
Source: J Spinal Cord Med. 2025 Mar 31;49(2):241–9. doi: 10.1080/10790268.2025.2479957 (PMC12931335; doi:10.1080/10790268.2025.2479957)
Supplement: Supplementary Material 1.docx [file YSCM_A_2479957_SM0199.docx]

**Supplementary Material 1**

*Analytic Steps*

Transcripts were analysed by the first and second authors ([initials removed for anonymous review]) to produce a coding framework with working codebook (inclusive of field notes, reflections and analytic memos). The first two steps of reflexive thematic analysis (familiarisation and generation of initial codes; Braun & Clarke, 2021), were used to code the first set of ten transcripts. The codes were then used to form a coding framework via an iterative process of stratified process coding, which was subsequently refined after application to each further set of five transcripts (following Saldana, 2021). The coding framework created a ‘living code book’ (3); when this approach is fused with the preliminary steps of thematic analysis (4), it enables interrogation of qualitative information at depth, maintaining heightened relevancy to the research aims. All transcripts were double-coded in full. The living codebook framework comprised 48 codes, developed iteratively to maximise clarity and transparency (2). On review with the research team, one code was removed: (i) *Mental health in Spinal Cord Injury Centres (SCIC)* as it was an isolated exploration of inpatient experiences and therefore outside of this study’s focus on the outpatient community. One further code was overweighted and therefore split in two to allow for the distinction between routes for accessing physical and mental health support, producing a framework consisting of 48 codes. Reflexive thematic analysis was then used to create the final overarching themes and a triangulation exercise completed to assess agreement between researchers regarding inclusion of codes and representative quotes within the themes (2). Sense checking was used to maximise the credibility of results by presenting themes to the Board of the Spinal Injuries Charity [name blinded for peer review], inclusive of people living with SCI (n = 5) or employees who were involved in delivery of mental health support as Healthcare Professionals (n = 5) (5). Final thematic wording and explanations were then reviewed by the research team after discussion with participants.

1. Braun V, Clarke V. Thematic Analysis [Internet]. SAGE; 2021 [cited 2022 May 5]. Available from: https://uk.sagepub.com/en-gb/eur/thematic-analysis/book248481

2. Saldana J. The Coding Manual for Qualitative Researchers. The Coding Manual for Qualitative Researchers. 2021;1–440.

3. Reyes V, Bogumil E, Welch LE. The Living Codebook: Documenting the Process of Qualitative Data Analysis. Sociological Methods & Research. 2021 Feb 8;0049124120986185.

4. Braun V, Clarke V. Conceptual and design thinking for thematic analysis. Qualitative Psychology. 2022 Feb;9(1):3–26.

5. Candela A. Exploring the Function of Member Checking. TQR [Internet]. 2019 Mar 24 [cited 2023 Apr 25]; Available from: https://nsuworks.nova.edu/tqr/vol24/iss3/14/
